# Intranasal administration of white tea alleviates the olfactory function deficit induced by chronic unpredictable mild stress

**DOI:** 10.1080/13880209.2020.1855213

**Published:** 2020-12-15

**Authors:** Wenhao Hu, Guixiang Xie, Tian Zhou, Jialu Tu, Jiayi Zhang, Zejie Lin, Haiyang Zhang, Liangcai Gao

**Affiliations:** a School of Life Science, East China Normal University, Shanghai, China; b Shanghai Key Laboratory of Regulatory Biology, Institute of Biomedical Sciences, School of Life Sciences, East China Normal University, Shanghai, China

**Keywords:** Mitochondria, behavioural experiment, OMP, BDNF

## Abstract

**Context:**

White tea [*Camellia sinensis* (L) O.Ktze. (Theaceae)] is popular in Asia, but its benefits on olfactory injury are unknown.

**Objective:**

The present study explores the effects of white tea on the olfactory injury caused by chronic unpredictable mild stress (CUMS).

**Materials and methods:**

C57BL/6J mice (WT) were exposed to CUMS. CUMS mice (CU) were intranasally treated with white tea extract [low tea (LT), 20 mg/kg; high tea (HT), 40 mg/kg] and fluoxetine (CF, 20 mg/kg) for 7 days. Several behavioural tests were conducted to assess depression and olfactory function. The transmission electron microscope (TEM) and semi-quantitative reverse transcription PCR were performed separately to observe the changes of related structures and genes transcription level.

**Results:**

The depressive behaviours of the LT and HT mice were reversed. The latency time of the buried food pellet test decreased from 280 s (CU) to 130 s (HT), while the olfactory sensitivity and olfactory avoidance test showed that the olfactory behaviours disorder of LT and HT mice were alleviated. The white tea increased the A_490 nm_ values of the cortisol treated cells from 0.15 to 1.4. Reduced mitochondrial and synaptic damage in the olfactory bulb (OB), enhanced expression of the brain-derived neurotrophic factor (BDNF) and olfactory marker protein (OMP) were observed in the LT and HT mice.

**Conclusions and discussion:**

White tea has the potential in curing the olfactory deficiency related to chronic stress. It lays the foundation for the development of new and reliable drug to improve olfactory.

## Introduction

The olfactory system is one of the oldest sensory systems and is crucial to the survival of animals. It has strong relevance in various behaviours like foraging and social behaviour (Breton-Provencher and Saghatelyan 2012). Dysfunctions of the olfactory system seriously affect health and the quality of life, such as reducing the enjoyment derived from food and the information received from the environment (Marin et al. 2018).

Since part of the olfactory system is constantly exposed to the environment, it is extremely vulnerable to damage. Particularly, the reduction of olfactory sensitivity is a clinical symptom of a variety of neuropsychiatric disorders, such as Alzheimer’s and Parkinson’s disease (Siopi et al. 2016). Anatomically, there is a partial overlap between the olfactory and limbic systems, suggesting that smell is involved in emotional processing. Through long-term observational and statistical studies, a large amount of evidence could explain the association between depression-like symptoms and olfactory. Studies have shown that the olfactory defect usually found in depressed patients is associated with the severity of clinical depressive symptoms (Croy et al. 2015), and the depressive symptoms of patients with congenital anosmia are much more enhanced (Croy et al. 2012). In addition, some studies have proven that transient olfactory deprivation induces mood and cognitive impairment in mice (Chen et al. 2019). All of these indicate that olfactory injury and depression have strong correlations.

Stress plays a major role in various physiological processes associated with neurodegenerative diseases and mental disorders (Esch et al. 2002). In particular, stress and depression are often experienced at the same time, which is quite common in contemporary society (Jaremka et al. 2013). The chronic unpredictable mild stress (CUMS) model is the most widely used depression model (Li et al. 2019). The olfactory system’s function and structure are impaired in depressed patients and depressed animal models, especially OB and olfactory epithelial cells (Yang et al. 2011).

Past research has revealed that the abundance of natural ingredients in food has health benefits for organisms. Examples include the hydroxytyrosol found in olives, resveratrol found in nuts and red wine, and dioscin found in yams, which have been shown to have antioxidant, antithrombotic, and anti-inflammatory properties (Kris-Etherton et al. 2002; Dai and Mumper 2010; Yang et al. 2017). Some herbs such as *Astragalus* (Theaceae), a precious medicinal dietary material rich in *Astragalus* polysaccharide, have the effect of biphasic regulation of blood glucose, and can effectively inhibit hyperglycaemia and complications in diabetic animal models (Li et al. 2004). Tea is one of the most widely consumed beverages in the world, as well as a kind of prescription. White tea, green tea, black tea, and puerh tea are the principal types of tea. They are all derived from the leaves of the tea plant [*Camellia sinensis* (L) O.Ktze. (Theaceae)] and are classified depending on the degree of oxidation of polyphenol.

Less attention has been paid to the health benefits of white tea. It is a simple processed tea (dried without premature wilting) and has similar or even better antioxidant activity than some green tea (Santana-Rios et al. 2001). White tea has been shown to contain more abundant anti-elastase, anti-ageing enzyme, and antioxidant activity than some green tea. Numerous studies have shown that white tea polyphenols can suppress the proliferation of colon cancer cell line HT-29 and protect the DNA of normal cells from oxidative damage *in vitro* (Hajiaghaalipour et al. 2015). It can also inhibit pancreatic lipase (Gondoin et al. 2010), and reduce fat accumulation in mice by increasing lipid metabolism and inhibiting inflammation (Liu et al. 2019). It may also enhance skin elasticity, reduce inflammation, and relieve rheumatoid arthritis (Thring et al. 2009).

Therefore, the present study investigated whether nasogastric white tea extracts could affect the olfactory ability of mice with lasting and unpredictable mild stress (CUMS). Additionally, our study provides theoretical support for the application of white tea for the olfactory disorder caused by exogenous chronic stress.

## Materials and method

### Animals

Fifty 6-week-old male C57BL/6J mice weighing 20–40 g were obtained from the Department of Experimental Animals (Fudan University, China). The animals were housed in standard polypropylene cages individually. All subjects were under constant temperature (22 °C) and 40–70% humidity, on a 12 h light/dark cycle light from 7:00am to 7:00pm, with access to food and water *ad libitum*. All experimental methods were approved by the Institutional Review Boards of East China Normal University. Procedures involving animals and their care were conducted in conformity with Directive 2010/63/EU of the European Parliament on the protection of animals used for scientific purposes and in accordance to ARRIVE guidelines. Our research was carried out in accordance with the National Institute of Health Guide for the Care and Use of Laboratory Animals (NIH Publications No.80-23) revised 1996 or the UK Animals (Scientific Procedures) Act 1986 and associated guidelines. We certify that formal approval to conduct the experiments described has been obtained from the animal subjects reviewed board of the Animal Ethics Committee of East China Normal University and could be provided upon request. All efforts were made to minimize the number of animals used and their suffering.

### Drug and administration

The white tea was purchased in March of 2018 at a tea factory in Fujian province, China, and authenticated by Prof. Hongqing Li, a botanist of East China Normal University. A voucher specimen of *Camellia sinensis* (Gao et al. 2018) was deposited in the HSNU, East China Normal University. According to the Chinese national standards of tea sensory evaluation, the dried leaves of white tea were ground to a powder and brewed with a leaf/extraction media ratio of 1:50 (w/w). The extraction medium was double distilled water. White tea leaves were processed at 85 °C for 10 min in a rotary evaporator in the extraction process. The mixture was removed by filtration and the infusion was quickly cooled to 30 °C. Then, the extract was diluted to low and high concentration using double distilled water and preserved at −20 °C for long-time storage. The drug was warmed to room temperature prior to feeding before intranasal administration.

Mice were randomly divided into five groups (*n* = 10) based on their test scores as follows: one control group (WT) and four tests groups, which offered low concentration white tea (LT, 20 mg/kg), high concentration white tea (HT, 40 mg/kg) and fluoxetine (CF, 20 mg/kg) as positive control and CUMS group (CU). When performing the intranasal delivery, mice were hand-restrained, positioned in a supine position, and the dose of 20 or 40 mg/kg for each mouse was administered (Daglia et al. 2017). Mice received extra treatment drop if the mouse expelled the solution. After delivery, an additional 5 s in the supine posture is necessary for mice to facilitate the delivery. The intraperitoneal dose of fluoxetine was 20 mg/kg.

### Chronic unpredictable mild stress procedure (CUMS)

**Figure 1.**
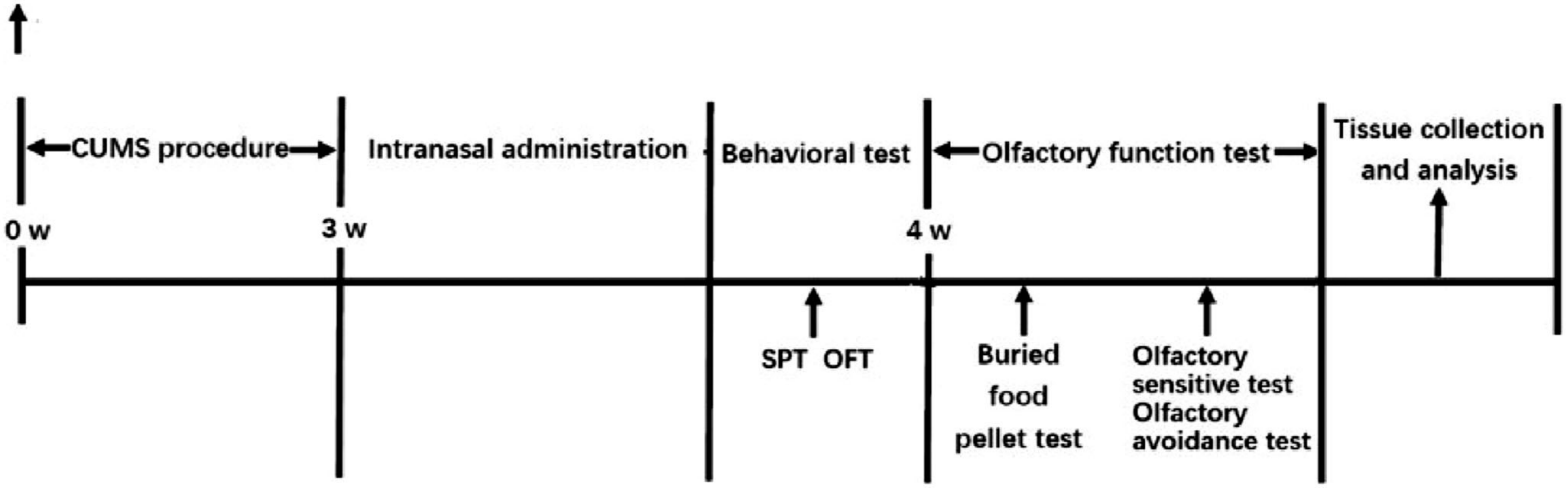
Schematic diagram of the experimental schedule. Mice were given the corresponding drug treatment in the last week of the 4-week chronic unpredictable mild stress (CUMS) exposure. The group of control mice was given no drug treatment in the 4-week general handling. OFT: open-field test; SPT: sucrose preference test.

Mice were exposed to CUMS for 4 weeks totally. The procedure of CUMS was performed as previously described (Huang et al. 2019), with minor modifications. In brief, the CUMS protocol consisted of various mild stressors: (1) food deprivation for 24 h, (2) water deprivation for 24 h, (3) cage tilt (45°) for 24 h, (4) inversion of the light/dark cycle, (5) soiled cage for 24 h, (6) forced swimming at 4 °C for 5 min, (7) tail pinching for 1 min. Stressors were arranged in a semi-random manner, at any time of day, in order to make the stress unpredictable. Control animals were housed in a separate room. After 3 weeks of CUMS, the animals of test groups were subjected to intranasal administration for 1 week while exposed to stress at the same time. After the nasal feed, all mice were subjected to various behavioural experiments. Mice were euthanized quickly 24 h after the last test. The olfactory bulbs were dissected and frozen at −80 °C for further biochemical analysis (Figure 1) for the flow chart of the specific experimental process.

### HPLC analysis of white tea extraction

The high-performance liquid chromatography (HPLC) analysis of the white tea was performed based on the previous study (Sunil et al. 2012). The extract of white tea was analyzed on an analytical Eclipse XDBC18 using a Luna C18 column (4.6 × 250 mm, 5 mm) with a flow rate of 1 mL/min and a sample size of 10 μL. The mobile phase was 0.06% methanol and phosphoric acid solution (methanol:phosphoric acid = 1:1). The results were detected by UV spectra on-line for peak identification from *λ* 190 nm to 400 nm. To prepare the standards, 5 mg of each reference substance [HPLC grade, catechin (C), epigallocatechin (EGC), gallocatechin (GC), and epigallocatechin gallate (EGCG)] were accurately weighed and dissolved in methanol (5 mL). These standard solutions were stored at −5 °C.

### Acute toxicity

Five groups of mice with 10 mice in each group orally received doses of 1, 2, 3 and 4 g/kg of extractions of white tea. Meanwhile, the control group was only given the vehicle (the double-distilled water). The mice were observed for 48 h and the number of deaths was counted at the end of this period. Lethal dose 50 (LD_50_) values were analyzed statistically and calculated by the logit method.

### Open-field test

Mice were individually placed in the middle of the open field (40 × 60 × 50 cm) with the floor of the arena divided into 25 equal squares. The number of squares crossed with all paws (crossing), was recorded during a period of 6 min (The first 1 min for mice to adapt and the last 5 min to record) using a camera. The floor of the open field apparatus was cleaned with 75% ethanol between tests.

### Sucrose preference test

All mice were trained to adapt to the sucrose solution: (1) 24 h exposure to two bottles of sucrose solution. (2) An additional 24 h exposure to one bottle of sucrose solution and one bottle of water. After that, the mice were deprived of food and water for 12 h. This test was conducted for 24 h, during which period the mice could freely access two bottles, one being 100 mL of water and the other being 100 mL of 1% (w/v) sucrose solution. The position of two bottles (left or right sides of the cages) was altered randomly. The measure was calculated using the following formula:

$$\text{The}\;\text{sucrose}\;\text{preference}\;\left(\%\right)=\frac{\left(\text{Sucrose}\;\text{intake}\right)}{\left(\text{Sucrose}\;\text{intake}\;+\;\text{Water}\;\text{intake}\right)}\;\times \;100\%.$$

### Buried food pellet test

The buried food pellet test was carried for two consecutive days. All the mice underwent food deprivation for 24 h before the test. Mice were examined only one trial per day. In each trial, a single mouse was placed in the middle of the test cage (42 × 35 × 40 cm^3^) to search for a 1 g food pellet which was buried approximately 0.5 cm below the surface of a 3 cm deep layer of the bedding material. The latency for each mouse to find the food pellet was identified as the time between when placing the mouse in the cage and when the mouse detected the food pellet and grasped it in its forepaws and/or teeth. If the mouse could locate the food pellet within 5 min, it could get back to its home cage with this pellet as a reward. Otherwise, no reward was given. The bedding in the test cage was changed between each trial and the location of the food pellet was changed daily in a random way. After finishing the test of the day, each mouse was given a 1 g food pellet to ensure its survival. The next day, repeated the procedure as the first day.

### Olfactory sensitivity test

The olfactory sensitivity test was conducted in a modification of the process described previously for mice (Witt et al. 2009). We used peanut butter as the source of the scent and the oil as the solvent. Multiple dilutions of the scent were made here, 0.1, 0.05, 0.01 g/mL, and pure oil, respectively. Besides, we also prepared filter paper (9 cm × 9 cm) for scent samples. First, we put the mice into a clean cage to adapt to the environment for 15 min. Then we switched the mouse to a uniform cage but with a piece of filter paper infiltrated one dilution for 3 min. The processes were recorded and analyzed by the Ethovision XT14 software package (Noldus IT, Leesburg, VA).

### Olfactory avoidance test

The olfactory avoidance test was similar to those of the olfactory preference test, but the test cage was divided into two parts (1:3) with a line drawing of the bottom of the cage. Mice moved freely in the test cage. The filter paper scented with test odorant was set into the smaller compartment. Odorant samples used were distilled water and 2-methylbutanoic acid (1.7 × 10^−6 ^mol) diluted by distilled water. Avoidance time was defined as the time spent in the area on the side of distilled water. The processes were recorded and analyzed by the Ethovision XT14 software package (Noldus IT, Leesburg, VA).

### Electron microscopic study

The olfactory bulbs were treated according to the procedure in previous studies (Gao et al. 2018). The olfactory bulbs were identified with a light microscope Leica MMAF (Hitachi High-Technologies Corporation, Beijing, China), and cut out from the coronal slices, dehydrated in a graded series of ethanol and acetone, and embedded in araldite. Blocks were trimmed and the sections were cut to the thickness of 70–75 nm with an ultramicrotome, after having double-stained with uranyl acetate and lead citrate, then examined with H7700 transmission electron microscopes (Hitachi High-Technologies Corporation, Beijing, China).

### Semi-quantitative reverse transcription-PCR

Total RNA was extracted from the olfactory bulbs using a Trizol reagent kit (Invitrogen, Carlsbad, CA). After evaluating the quantity and quality of purified RNA, 2000 ng of total RNA samples of each group was used for RT-PCR reaction. cDNA synthesis was carried out using RNA PCR Kit (AMV, Takara, Japan). Two real-time primers were used for amplification. The primers for the BDNF forward: 5′-TCATACTTCGGTTGCATGAAGG-3′ and reverse: 5′-AGACCTCTCGA ACCTGCCC-3′ and the primers for the olfactory marker protein forward: 5′-CAGCAGGAAGGTTCTCCTCC-3′ and reverse: 5′-GAACAGCCAGGATATGCCCA-3′ were designed by the National Centre for Biotechnology Information (NCBI) and synthesized by cooperation with Sangon Biotech (Shanghai) Co., Ltd. (Shanghai, China). The PCR condition was: 5 min at 94 °C (one cycle), 30 s at 94 °C, 30 s at 58 °C, 30 s at 72 °C (30 cycles), and 10 min at 72 °C (one cycle). 18srRNA gene is used for internal control and measures the expression level of the target gene. The PCR products were analyzed by agarose gel electrophoresis. Semi-qualified and analyzed the densities of the electrophoresed PCR product by software Image J (bundled with 64-bit Java 1.8.0_112).

### SH-SY5Y cells culture and evaluation of cell viability

The SH-SY5Y cells were purchased from ATCC. Cells (2 × 10^5^ cells per mL of media) 200 μL were seeded in 96-well microplates and incubated for 24 h (37 °C, 5% CO_2_ air humidified). The cells were infiltrated with serum-free DMEM containing 10 μM cortisol for 48 h, a blank control group was also set. Then, the cells were incubated with white tea extracts (10 μM) and fluoxetine was used as a positive control (10 μM) for 24 h. To evaluate cell survival, 20 μL of MTT solution (5 mg/mL) was added to each well and incubated for 4 h. Then, the DMSO solution was taken place of the MTT. Absorbance was determined at 490 nm by an ELISA plate reader.

### Statistical analysis

All results are presented as mean ± SEM. The data were analyzed using the GraphPad Prism (version 7.0, San Diego, CA. One-way and two-way analysis of variance (ANOVA) were used for data analysis. In each analysis, significant main effects were followed by Bonferroni’s *post hoc* test. *p* < 0.05 was considered statistically significant.

## Results

### HPLC analysis of white tea extraction

The white tea extracts analysis showed that epigallocatechin gallate (EGCG) is the most active compound of white tea (EGCG, 60.25%), followed by epigallocatechin (EGC; 3.12%), gallocatechin (GC; 1.26%), and catechin (C; 0.69%) which were also found in the extract.

### The toxicity of white tea extraction

The oral administration of white tea extraction up to the doses of 4 g/kg showed no obvious signs of acute toxicity during 48 h of observation. Therefore, LD_50_ could not be determined. The result of the pharmacological tests performed indicated that white tea is of low toxicity.

### The effect of white tea extracts on open field test

**Figure 2.**
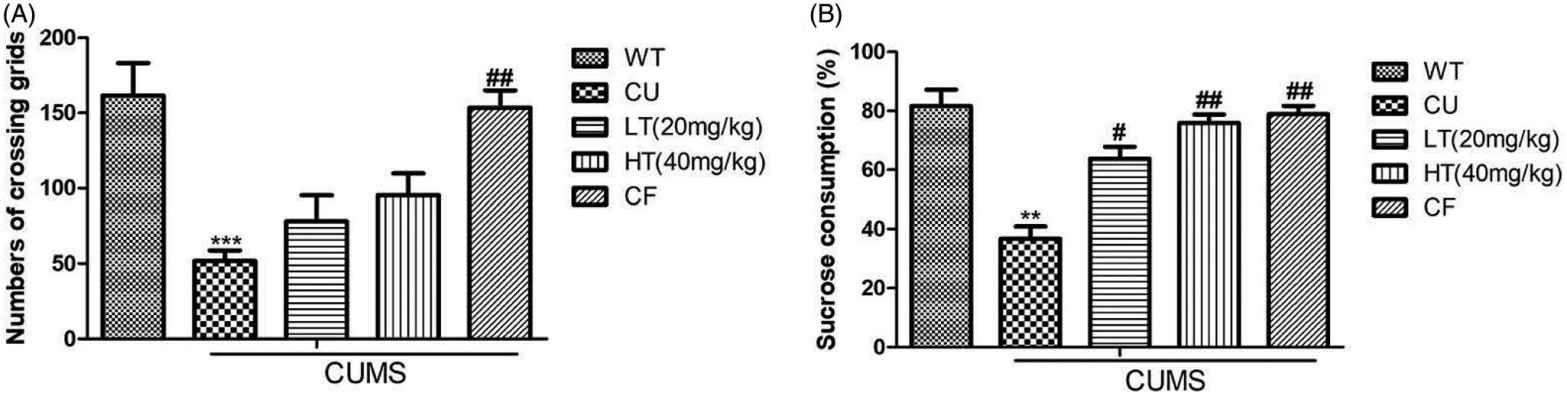
Effects of intranasal administration of white tea extracts on the open field test and sucrose preference test. (A) Latency time of five groups of open field test (OFT) after 3 weeks CUMS. (B) Sucrose preferences of the five groups after 3 weeks CUMS. Data are expressed as means ± SEM (*n* = 10). **p < 0.01 vs. control group; #*p* < 0.05 vs. CUMS group; ##*p* < 0.01 vs. CUMS group.

Travelling distance in the open field can be utilized to judge whether mice are under anxiety. Mice were tested in the open field test apparatus to record their crossing number. One-way ANOVA showed a significant decline in travelling distance in CU mice compared to WT mice (Figure 2(A); *p* < 0.001). However, LT and HT mice showed no obvious difference to CU mice.

### The effect of white tea extracts on the sucrose preference test

The consumption of sucrose is another indicator to measure depression-like behaviours. CU mice showed a dramatic reduction in relative sucrose intake compared with the WT mice (Figure 2(B); *p* < 0.01), while the drug treatment group presented effects of intranasal administration of white tea significantly in SPT (LT: *p* < 0.05; HT: *p* < 0.01).

### The effects of white tea extraction on the food buried test

**Figure 3.**
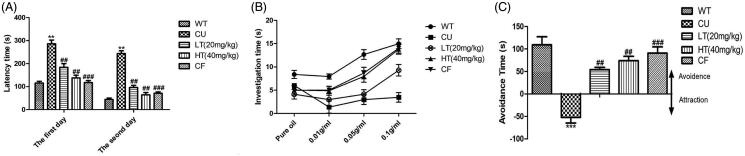
Effects of intranasal administration of white tea extracts on the general olfactory functions of CUMS-induced mice. Mice performed the (A) buried food pellet test, (B) olfactory sensitive test and (C) olfactory avoidance test after the intranasal administration. PB: peanut butter. Data are expressed as means ± SEM (*n* = 10). **p < 0.01 vs. control group; ##*p* < 0.01 vs. CUMS group.

Buried food pellet test is considered as a standard method to investigate the olfactory ability of mice related to food. Mice with different treatments all found the pellets more rapidly in the next day (Figure 3(A); WT: *p* < 0.001; CU: *p* < 0.05; LT: *p* < 0.01; HT: *p* < 0.01), but CU mice were significantly slower at finding buried pellets than WT mice in two days (the first day, *p* < 0.01; the second day, *p* < 0.01). Treatments to the mice of CUMS, LT and HT, decreased the latency significantly in two day tests. (The first day: LT: *p* < 0.05; HT: *p* < 0.01; the second day: LT: *p* < 0.05; HT: *p* < 0.01).

### The effects of white tea extraction on the olfactory sensitive test

The two-way ANOVA with the concentration of peanut butter (PB) and treatment as factors was performed. When testing the scent of pure oil, there was no significance among all groups. CU mice showed fewer interests than control mice significantly (Figure 3(B); 0.01 g/mL PB, *p* < 0.001; 0.05 g/mL PB, *p* < 0.001; 0.1 g/mL PB, *p* < 0.001, respectively). LT mice increased the investigation time significantly only for 0.1 g/mL PB (*p* < 0.001). However, HT mice had markedly increase of investigation time for 0.05 and 0.1 g/mL PB (0.05 g/mL PB, *p* < 0.05; 0.1 g/mL PB, *p* < 0.01).

### The effects of white tea extracts on olfactory avoidance test

When investigating whether mice differed in their ability to avoid aversive odorants in the olfactory avoidance test (Figure 3(C)). CU mice did not avoid the odour of 2-methylbutanoic acid contrasted with WT mice (*p* < 0.001). LT and HT groups increased the avoidance time compared to CU mice significantly (LT: *p* < 0.01; HT: *p* < 0.01).

### Intranasal white tea extracts rescue the mitochondrial and synaptic damage in the olfactory bulb

**Figure 4.**
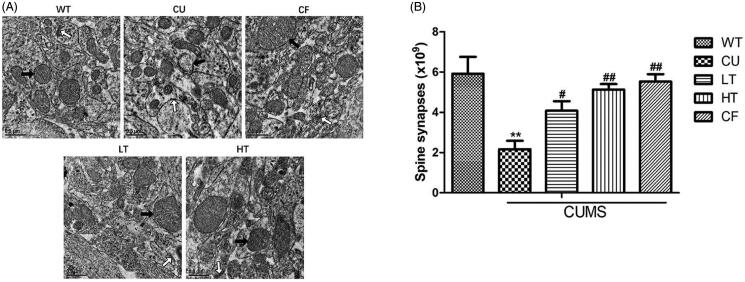
Effects of intranasal administration of white tea extracts on the structure of mitochondria and the number of spine synapses in olfactory bulb cells. (A)The photomicrograph by transmission electron microscopy (TEM) is representative of mitochondria and synapses in olfactory bulb cells from each experimental group. The black arrows mark the mitochondria and the white arrows mark the synapses. (B) Asymmetric spine synapses were counted according to the rules of the dissector technique within an unbiased counting frame superimposed onto each electron micrograph. The average volumetric density (synapse/μm^3^) of spine synapses within each sampling area was then determined by dividing the sum of spine synapses counted in all samples taken from that particular sampling area by the dissector volume. Finally, the volumetric density of spine synapses was multiplied by the volume of the sampling area to arrive at the total estimated number of spine synapses. Data are expressed as means ± SEM (*n* = 10). **p < 0.01 vs. WT group; #*p* < 0.05 vs. CU group; ##*p* < 0.01 vs. CU group; ###*p* < 0.001 vs. CU group.

Mitochondrial morphology and synapse damage are features of neuroplasticity in olfactory bulb cells. All groups’ olfactory bulbs were investigated using transmission electron microscopy (Figure 4(A,B)). In CU mice’s olfactory bulbs, disruption of cristae, vacuolar degeneration, and even interruption of the mitochondrial membrane were detected. Compared with CU mice, mitochondria of LT and HT groups had relatively less vacuolar degeneration and more intact cristae. Moreover, the synaptic gaps of the LT and HT groups were denser and vesicles were more than those of the CU group. The number of spine synapses in the olfactory bulb (Figure 4(B)) of five groups was calculated. One-way ANOVA demonstrated conspicuous fewer spine synapses in CU mice (*p* < 0.01). LT and HT mice actually increased significantly (LT: *p* < 0.05; HT: *p* < 0.01).

### Intranasal white tea extracts recover the brain-derived neurotrophic factor and olfactory marker protein

**Figure 5.**
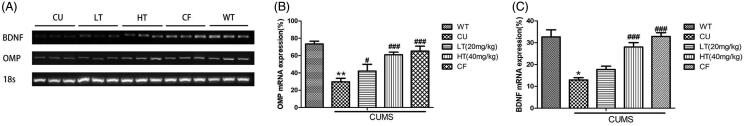
Effects of intranasal administration of white tea extracts on the mRNA expression level of OMP and BDNF in the olfactory bulb. The mRNA expression in the olfactory bulbs of OMP and BDNF affected by white tea extracts. (A) The agarose gel electrophoresis result of OMP and BDNF. (B) The relative mRNA expression level of OMP. (C) The relative mRNA expression level of BDNF. Data are expressed as means ± SEM (*n* = 10). **p < 0.01 vs. WT group; #*p* < 0.05 vs. CU group; ##*p* < 0.01 vs. CU group. ###*p* < 0.001 vs. CU group.

Bdnf and Omp mRNA expression in the olfactory bulb were detected by semi-quantitative RT-PCR to further investigate olfactory injury in CUMS mice and changes of related molecules after nasal feeding white tea extracts (Figure 5(A,B)). OMP mRNA expression of mice in the CU group was significantly decreased compared with that in the control group (Figure 5(B); *p* < 0.01), while both the LT and HT groups showed significantly increased expression compared with the CU group (LT: *p* < 0.05; HT: *p* < 0.001). BDNF showed a similar change as OMP expression (Figure 5(C); *p* < 0.05). However, no significant difference was found between the LT group and CU group while a significant difference appears between the HT group and CU group (*p* < 0.001).

### Effects of white tea extracts on SH-SY5Y cells from the lesion induced by corticosterone

**Figure 6.**
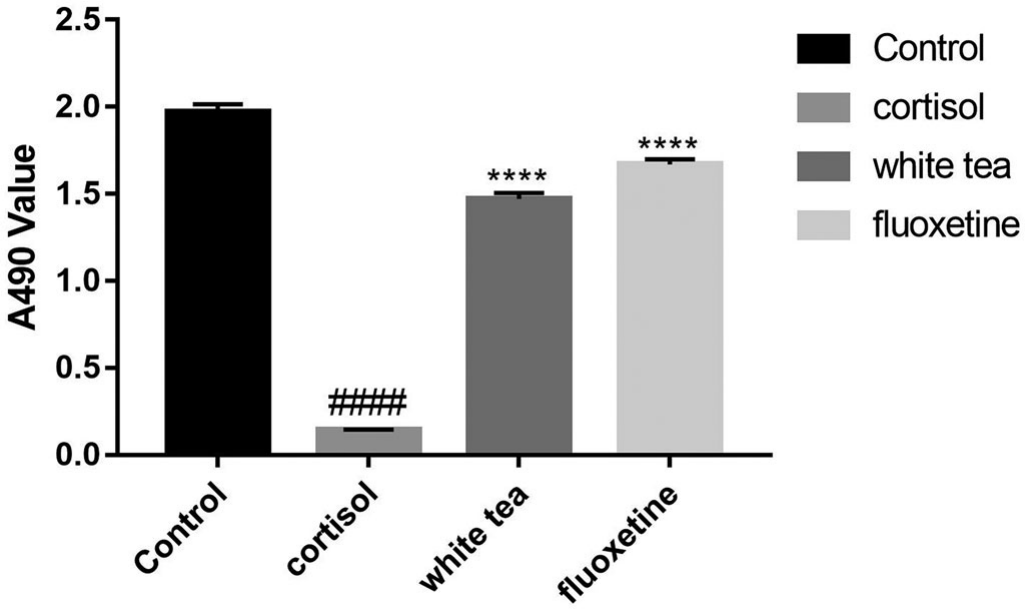
Effects of white tea extracts on SH-SY5Y cells from the lesion induced by corticosterone. The cell viability after treated with white tea and fluoxetine was tested by MTT tests. Data are expressed as means ± SEM (*n* = 12). ****p < 0.0001 vs. cortisol group; ####*p* < 0.0001 vs. control group.

After SH-SY5Y cells were treated with cortisol for 48 h, the A_490nm_ values decreased significantly compared with control (Figure 6; *p* < 0.0001). Then, the cells were incubated with white tea extracts and fluoxetine. Results showed that both treatments reversed the damage to the cells markedly (Figure 6; *p* < 0.0001).

## Discussion

In the present study, after mice receiving CUMS, the state of anxiety was measured by the OFT and SPT experiments. However, no marked changes can be seen in the OFT after giving white tea while a significant increase in sucrose consumption occurred in both LT and HT mice, which indicates that white tea extracts could release mice’s anxiety state without enhancing their locomotive ability. From olfactory experiments, we can see that intranasal white tea significantly improved the decline of olfactory function in CUMS mice. Next, we further reveal that white tea mitigated olfactory damage caused by CUMS of morphological and molecular levels. Through observing the structure of the olfaction bulb, we demonstrated that white tea extracts have a good effect on improving mitochondrial structural damage, such as cristae fracture and vacuolar degeneration. In addition, the number of synapses and the state of synaptic vesicles after nasal feeding were also quite similar to those of the positive control group. At the molecular level, the mRNA expression level of OMP in the olfactory bulb was markedly increased after white tea was given, and the expression level of BDNF showed a close trend to that of OMP. Besides, we also conducted cell viability tests, results indicated that white tea extracts have a considerable protective ability as fluoxetine and showed weak toxic effects on the cells. These results all suggested that white tea could improve the olfactory damage caused by CUMS by repairing the structure and function of olfactory sensory neurons.

Due to exceptional antioxidant capacity, white tea has been proved to have a relief effect on metabolic imbalance and oxidative stress associated with diabetes (Xu et al. 2017). In addition, it has also been proved to have an anti-mutation effect (Santana-Rios et al. 2001) and a protective effect on neurotoxicity caused by oxidative stress (Daglia et al. 2017). Depending on previous studies, white tea is rich in catechins, such as EGC and GCG (Nunes et al. 2015). These compounds are effective in scavenging reactive oxygen species (ROS) (Almajano et al. 2011) and have metal-ion chelating functions, which can alleviate the problem of increased erythrocyte malondialdehyde (MDA) levels and decreased GSH activity in aging mice (Maurya and Rizvi 2009). Because the processing is relatively simple, the compounds are more abundant than other types of tea. Containing more active constituent, the white tea extracts at the concentration of 40 mg/kg showed comparable effects as the positive control drug in our study.

Unlike traditional methods, we used nasogastric to deliver our extracts. This method builds on the anatomical characteristics of the olfactory system, which enters the central nervous system without bypassing the blood–brain barrier blocking (Cheng et al. 2016). Olfactory sensory neurons of the dendritic are directly exposed to the external environment, while their axons via the ethmoid bone are projected onto the olfactory bulbs. Intranasal injection of insulin provides a rapid insulin delivery to the central nervous system through the large flow of olfactory and perivascular channels of the trigeminal nerve, achieving better results than the traditional administration. Herein, CUMS was used to treat mice, and the repair of olfactory damage was observed, showing a very ideal effect.

We used the classic stress model, the CUMS mice in this study. This model has been widely used to assess depression-like behaviour in mice and rats. Some previous studies mentioned that CUMS mice and rats’ models significantly alleviated depression-like behaviour after drug administration (Li et al. 2018; Liu et al. 2018). Further experiments also showed that Chinese herbal medicine can correct the HPA axis dysfunction and inhibit the occurrence of inflammation (Cai et al. 2015; Wang et al. 2018; Wu et al. 2019). In addition, previous studies have shown that an olfactory bulb and olfactory epithelium in CUMS mice may be affected by two pathways: neural and energy metabolism (Abd El-Fattah et al. 2018). Along with the performance in behavioural experiments, the mitochondria in CUMS mice appeared serious cavitation and the breakage of cristae. Mitochondria participate in many important metabolic processes like the Krebs cycle. Besides, it is likewise the main source of reactive oxygen species (ROS). Oxidative stress occurs not part of the chronically stressed mice, but systemically (Luo et al. 2010). The ruin of the structure usually means a decline function. Besides, the density of synapses indicates the connection between neurons to some extent. As the storage places and release sites of neurotransmitters, the synaptic vesicles are closely linked to the state of neurons (Leenders et al. 1999), reflecting the activity of neurons in the olfactory bulbs.

In addition, mRNA expression levels of OMP, which is the marker of mature olfactory neurons, displayed a remarkable recovery after white tea feeding. BDNF showed a similar change in OB, which is consistent with the clinical presentation of depressed patients (Zimmerberg et al. 2009). Previous results have shown that BDNF is involved in the regeneration of OB olfactory receptor neurons and BDNF may play a critical role in neonatal rats’ olfactory association learning (Zhang et al. 2018). Further studies also proved a close relationship between BDNF and mitochondria. It is believed that oxidative stress caused dysfunction in mitochondria and reduces the level of BDNF (Ye et al. 2012). BDNF has been shown to improve the respiratory efficiency of brain mitochondria (Markham et al. 2004). Combined with the observed structural repair of mitochondria, it can be assumed that the mechanism of action of white tea may be closely related to the above process.

As noted above, our study demonstrated that intranasal white tea extracts can help relieve olfactory functional damages caused by CUMS. This process may involve in the interaction among the mitochondria, BDNF, and oxidative stress. However, the detailed functional mechanisms of white tea during olfactory recovery after CUMS need to be investigated in further studies.

## Author contributions

W.H. and G.X. designed the study; W.H, T.Z., G.X., H.Z., J.T. performed the study; W.H., T.Z. and G.X. analyzed the behavioural test data; W.H, T.Z., and G.X. drafted the manuscript. J.T. and J.Z. revised the manuscript. Z.L. provided technical assistance. All authors read and approved the manuscript.
